# Biological evaluation of antibody-maytansinoid conjugates as a strategy of RON targeted drug delivery for treatment of non-small cell lung cancer

**DOI:** 10.1186/s13046-016-0347-6

**Published:** 2016-04-22

**Authors:** Liang Feng, Hang-Ping Yao, Yong-Qing Zhou, Jianwei Zhou, Ruiwen Zhang, Ming-Hai Wang

**Affiliations:** State Key Laboratory for Diagnosis & Treatment of Infectious Diseases and Collaborative Innovation Center for Diagnosis & Treatment of Infectious Diseases, First Hospital of Zhejiang University School of Medicine, Zhejiang, China; Department of Biomedical Sciences, Texas Tech University Health Sciences Center School of Pharmacy, 1406 Coulter Street, Suite 1117, Amarillo, TX 79106 USA; Department of Neurosurgery, First Hospital of Zhejiang University School of Medicine, Zhejiang, China; Department of Molecular Cell Biology & Toxicology, Nanjing Medical University School of Public Health, Jiangsu, China; Department of Pharmaceutical Sciences, Texas Tech University Health Sciences Center School of Pharmacy, Amarillo, TX USA

**Keywords:** Antibody-drug conjugate, Breast cancer, Lung cancer, Receptor tyrosine kinase, Therapeutic efficacy, Combination therapy

## Abstract

**Background:**

Aberrant expression of the RON receptor tyrosine kinase, a member of the MET proto-oncogene family, in breast cancer and non-small cell lung cancer (NSCLC) has therapeutic implication. Here we evaluated the efficacy of a novel anti-RON antibody-drug maytansinoid conjugate Zt/g4-DM1 for treatment of breast and NSCLC xenograft tumors in mouse models and explored a treatment strategy by combination of Zt/g4-DM1 with chemotherapeutics to achieve the maximal therapeutic activity.

**Methods:**

Mouse monoclonal antibody Zt/g4 (IgG1a/κ) specific to human RON was conjugated to DM1 via thioether linkage to form Zt/g4-DM1 with a drug-antibody ratio of 4:1. Several breast cancer and NSCLC cell lines, expressing different levels of RON, were used as the model. Immunofluorescence was used to determine Zt/g4-induced RON internalization. Flow cytometric analysis and cell viability assay were used to determine the effect of Zt-g4-DM1 on cell cycle and death. Mouse xenograft NSCLC models were used in vivo to determine the therapeutic efficacy of Zt/g4-DM1 alone or in combination with chemotherapeutics.

**Results:**

In vitro, Zt/g4 treatment of breast cancer and NSCLC cells rapidly induced cell surface RON internalization, which results in intracellular delivery of DM1 sufficient to arrest cell cycle at G2/M phase, reduce cell viability, and cause massive cell death. In mouse tumor xenograft models, Zt/g4-DM1 at 20 mg/kg in a Q12 × 2 regimen effectively blocked breast cancer and NSCLC cell- mediated tumor growth. More than 95 % inhibition of tumor growth among three tumor xenograft models tested was achieved according to the measured tumor volume. The minimal dose to balance the tumor growth and inhibition (tumoristatic concentration) was established at 2.02 mg/kg for H2228, 1.94 mg/kg for H358 cell, and 6.25 mg/kg for T-47D cell-mediated xenograft tumors.

**Conclusion:**

Zt/g4 is highly effective in RON-directed drug delivery for targeted inhibition of NSCLC cell-derived tumor growth in mouse xenograft models. This work provides the basis for clinical development of humanized Zt/g4-DM1 for potential cancer therapy in the future.

## Background

Antibody-directed drug delivery in the form of antibody-drug conjugates (ADC) is a promising anti-cancer strategy [1–3]. Structurally, ADC is formed by a target-specific monoclonal antibody (mAb) conjugated with a highly potent toxic drug through a versatile linker at a precise drug- antibody ratio [1–3]. Since 2012, two ADCs, ado-trastuzumab emtansine (T-DM1, Kadcyla, Roche-Genentech) and brentuximab vedotin (SGN-35, Adcetris, Seattle Genetics) have been approved for targeted therapy of breast cancer (BC) and lymphomas, respectively [4–7]. T-DM1 is specific to HER2 expressed by BC [4]. SGN-35 targets the CD30 antigen, a cell surface antigen expressed by Hodgkin’s and other subsets of lymphoma cells [6, 7]. Currently, ADCs employing different antibodies targeting different cell surface proteins such as PSMA [8, 9]. Ephrin-A4 [10], CD78B [11, 12], Trop-2 [13], and LY6E [14] are under intensive study in preclinical models. More than 30 ADCs are under clinical trials (www.clinicaltrials.gov). Thus, ADCs represent a promising therapeutic modality for the clinical management of cancer.

The RON receptor tyrosine kinase is a member of the MET proto-oncogene family implicated in the pathogenesis of BC and non-small cell lung cancer (NSCLC) [15–20]. Immunohistochemical staining using specific antibodies has shown increased RON expression in primary BC and NSCLC samples [21–24], which results in aberrant signaling facilitating cancerous cell growth, migration, and invasion [16]. Overexpression of RON also has been considered as a potential drug target for cancer treatment [25–29]. Currently, RON-specific therapeutics including tyrosine kinase inhibitors (TKI) and therapeutic monoclonal antibodies (TMA) have been developed and validated in various preclinical cancer models [25–29]. Clinical trials using RON-specific TKIs and TMAs are also under investigation (www.clinicaltrials.gov). Evidence accumulated from various studies showed that inhibition of RON has the therapeutic effects on cancer cell growth, migration, and survival [25–29]. However, the efficacy of RON- specific TKIs and TMAs is relatively low with only partial tumor inhibition [25–29]. The inability of anti-RON TKIs and TMAs to achieve the maximal effect is mainly due to the lack of complete addiction of cancer cells to RON signaling for growth and survival [16]. Thus, it is critical to improve the drug efficacy for the success of RON-targeted cancer therapy.

For the last several years, we have focused on anti-RON mAb-directed drug delivery for targeted cancer therapy [25, 30–32]. Several mAbs specific to human RON were produced and selected for their suitability for RON-targeted drug delivery [25, 30–32]. Zt/g4 is one of the anti- RON mAbs chosen for RON-targeted drug delivery [25, 33, 34]. Zt/g4 recognizes an antigenic epitope located in the Sema domain of the RON extracellular sequence and induces rapid internalization of the cell surface RON molecules [25, 33, 34]. This property makes Zt/g4 a suitable candidate to conjugate with DM1 to form anti-RON ADC Zt/g4-DM1 for targeted drug delivery. In this study, we used BC and NSCLC cells as the experimental models to determine the effectiveness of Zt/g4 in induction of RON internalization by a panel of BC and NSCLC cell lines expressing different levels of RON. The cytotoxic activity of anti-RON ADC Zt/g4-DM1 was evaluated in both in vitro cell lines and in vivo xenograft tumor models. Moreover, we explored a treatment strategy by combination of Zt/g4-DM1 with chemotherapeutics to achieve the maximal therapeutic activity against BC and NSCLC xenograft tumors. We believe that results from these studies confirm the potentials of anti-RON ADC for BC and NSCLC treatment and establish the foundation for development of humanized anti-RON ADC.

## Methods

### Cell lines and reagents

Human BC cell lines MCF-7, DU4475, MDA-MB-231, T-47D, and NSCLC cell lines H1993, H2228, and H358 were from ATCC (Manassas, VA). Mouse anti-RON mAbs Zt/g4, Zt/c1 and rabbit anti-RON IgG antibodies were as previously described [33]. Goat anti-mouse IgG labeled with FITC or rhodamine was from Jackson ImmunoResearch (West Grove, PA).

### Generation of Zt/g4-DM1

Zt/g4 was conjugated with DM1 at a drug-antibody ratio of 4:1 by through the linker SMCC as previously described [1, 4, 25, 35]. Control mouse IgG conjugated with DM1 (CmIgG-DM1) was used as the control. The Zt/g4-DM1 was verified by HIC using a Varian Prostar 210 Quaternary HPLC system coupled with a TSK butyl-NPR 4.6 × 3,5 column (Tosoh Biosciences (Prussia, PA) [25].

### Assay for cell surface RON expression

Cell surface RON was quantitatively determined by the immunofluorescence assay using QIFKIT® reagents from DAKO (Carpentaria, CA). Briefly, cells (1 × 10^6^ cells per ml in PBS) were treated with Zt/g4 at saturating concentrations followed by incubation in parallel with the QIFIKIT® beads and goat F(ab’)_2_ F0479. After establishing a calibration curve, the number of RON receptor on the cell surface was determined by interpolation following the manufacturer’s instruction.

### Analysis of cell cycle

BC and NSCLC cells (1 × 10^6^ cells per dish) were treated with 5 μg/ml of Zt/g4-DM1 at 37 °C for 24 h. Cells were then labeled with propidium iodide followed by Accuri flow cytometric analysis. Cell cycle changes were determined by measuring DNA contents as previously described [36].

### Western blot analysis

Cellular proteins (50 μg per sample) were separated in an 8 % SDS- PAGE under reduced conditions. Cellular proteins such as PARP were detected in Western blotting using specific antibodies. Membranes also were reprobed with anti-actin antibody to ensure equal sample loading [31].

### Detection of intracellular RON

BC and NSCLC cells at 1 × 10^5^ cells per well in a 6-well plate were treated with 5 μg/ml of Zt/g4-DM1 for various times followed by goat anti-mouse IgG coupled with FITC [35]. Nuclear DNAs were stained with 4′,6-diamidino-2-phenylindole (DAPI) [36]. Cellular immunofluorescence was observed under an Olympus microscope equipped with DUS/fluorescent apparatus.

### Assays for cell viability and cell death

Cell viability 96 h after Zt/g4-DM1 treatment was determined by the MTT assay [31, 36]. Percentages of viable or dead cells were determined using the trypan blue exclusion assay as previously described [31, 36].

### Mouse xenograft tumor model and anti-RON ADC treatment

All experiments on mice were approved by the TTUHSC Institutional Animal Care Committee. Female athymic nude mice at 6 weeks of age (Taconic, Cranbury, NJ) were injected with 5 × 10^6^ cancer cells in the subcutaneous space of the right flank as previously described [25, 33]. Mice were randomized into different groups (five mice per group). Treatment began when tumors reaches a mean tumor volume of ~100 mm^3^. To evaluate the Zt/g4-DM1 efficacy, mice were injected with Zt/g4- DM1 at 20 mg/kg in a Q12 × 2 regimen. Tumor growth was observed up to 52 days. In study of Zt/g4-DM1 in combination with gemcitabine, tumor-bearing mice were treated with gemcitabine alone at 60 mg/kg in a Q4 × 4 schedule [37], Zt/g4-DM1 alone at 10 mg/kg in a Q8 × 2 schedule, or their combinations with above described concentrations. Tumor volumes were measured every 4 days and calculated as previously described [24, 25]. Animals were euthanized when tumor volumes exceeded 2000 mm^3^ or if tumors became necrotic or ulcerated through the skin.

### Statistical analysis

GraphPad 6 software was used for statistical analysis. Results are shown as mean ± SD. The data between control and experimental groups were compared using Student *t* test. Chi-squared analysis was used for correlational study. Isobolograms were used for analysis of synergism in drug combination studies. Statistical differences at *p* <0.05 were considered significant.

## Results

### Induction by Zt/g4-DM1 of cell surface RON internalization

**Fig. 1:**
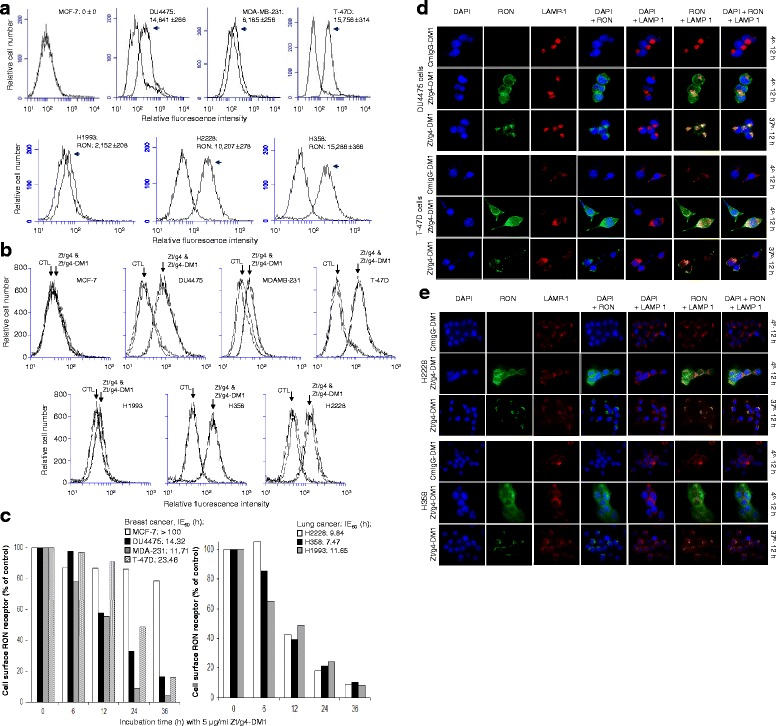
Binding and induction of RON internalization by Zt/g4-DM1. **a** Levels of RON expression by BC and NSCLC cell lines. Individual BC and NSCLC cell lines (1 × 10^6^ cells/ml) in 1 ml PBS in duplicates were incubated at 4 °C with 5 μg/ml of Zt/g4 for 60 min. Isotope matched mouse IgG was used as the control. Cell surface RON was quantitatively determined by immuno-fluorescence analysis using QIFKIT® (DAKO). The number of RON receptors was in a single cell was calculated according to the DAKO’s instruction. **b** Binding of DM1-conjugated Zt/g4 to cell surface RON. Individual BC or NSCLC cell lines at (1 × 10^6^ cells/ml) were incubated at 4 °C with 5 μg/ml of Zt/g4-DM1 for 60 min followed by flow cytometric analysis. Control mouse IgG (CTL) and free Zt/g4 were used as the control. **c** The time-dependent RON internalization. BC and NSCLC cells (1 × 10^6^ cells per dish) were treated at 37 °C with 5 μg/ml of Zt/g4-DM1, collected at different time points, washed with acidic buffer to remove Zt/g4 bound on the cell surface (31), and then incubated with 2 μg/mL of anti-RON mAb 2F2 [23]. Immunofluorescence was analyzed by flow cytometer using FITC-coupled anti-mouse IgG. The FITC-binding intensity from cells treated with Zt/g4-DM1 at 4 °C was set as 100 %. The IE_50_ values were calculated as the time required to achieving 50 % reduction of cell surface RON. **d** and **e** Immunofluorescent analysis of cytoplasmic RON: BC and NSCLC cells (1 × 10^5^ cells per chamber) were treated at 4 °C or 37 °C with 5 μg/ml of Zt/g4-DM1 for 12 h followed by FITC-coupled anti-mouse IgG. CmIgG-DM1 was used as the control. After cell fixation, immunofluorescence was detected using the BK70 Olympus microscope equipped with a fluorescence apparatus. LAMP1 was used as a marker for protein cytoplasmic localization. DAPI was used to stain nuclear DNA

To study the effect of Zt/g4 on RON internalization, we first determined the number of RON molecules expressed on cell surface using the QIFKIT® fluorescence-based quantitative method (Fig. 1a). The calculated RON molecules on the surface of a single cell was 14,841 ± 266 for DU4475, 8185 ± 256 for MDA-MB231, 15,756 ± 314 for T-47D, 2152 ± 208 for H1993, 10,207 ± 278 for H2228, and 15,286 ± 366 for H358 cells, respectively. Specific binding was not observed in MCF-7 cells. The binding profiles of DM1-conjugated Zt/g4 were shown in Fig. 1b. Mouse IgG and its DM1 conjugates (CmIgG-DM1) were used as the control. When antibodies were used at 5 μg IgG per ml, the RON binding profile of Zt/g4-DM1 was similar to that of free Zt/g4 among seven cell lines tested, suggesting that DM1 conjugation does not impair the binding capability of Zt/g4.

The effect of Zt/g4-DM1 on RON internalization is shown in Fig. 1c. Zt/g4-DM1 treatment caused a progressive reduction of cell surface RON in a time-dependent manner in all six cell lines tested. Less than 20 % of RON remained on the cell surface after a 36 h treatment. The effect of Zt/g4-DM1 on RON expressed by MCF-7 cells was minimal. We defined the time required to have a 50 % reduction in cell surface RON as the internalization efficacy (IE_50_). The calculated IE_50_ values were >100 h for MCF-7, 14.32 h for DU4475, 11.71 h for MDA- MB-231, 23.46 h for T-47D, 11.65 h for H1993, 7.47 h for H358, and 9.84 h for H2228 cells (Fig. 1c). These results indicate that Zt/g4-DM1 differentially induces RON internalization in different cancer cells. Immunofluorescence analysis confirmed Zt/g4-DM1-induced RON internalization in four selected cell lines (Fig. 1d and e). RON was detected on the cell surface at 4 °C. The internalization occurred at 37 °C after Zt/g4-DM1 treatment. Cytoplasmic RON was co-localized with LAMP1 in both BC and NSCLC cells. Thus, results from Fig. 1 demonstrate that Zt/g4-DM1 effectively induces RON internalization by BC and NSCLC cells.

### Effect of Zt/g4-DM1 on cell cycle, growth, and death of BC and NSCLC cells

**Fig. 2:**
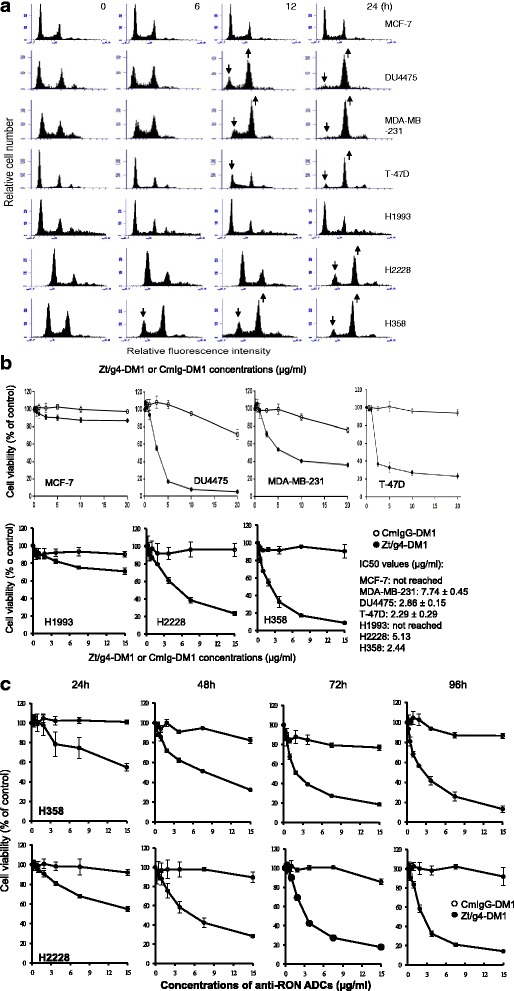
Effect of Zt/g4-DM1 on BC and NSCLC cell cycle, survival, and death. **a** Cell cycle changes: BC and NSCLC cell lines (1 × 10^6^ cells per dish) were treated at 37 °C with 5 μg/ml of Zt/g4-DM1 for various times, collected, stained with propidium iodide, and then analyzed by flow cytometer as previously described [36]. MCF-7 cells were used as the control. Changes in cell cycle were marked with arrows. **b** Reduction of cell viability: BC and NSCLC cell lines (5000 cells per well in a 96-well plate in triplicate) were treated with different amounts of Zt/g4-DM1 for 96 h. Cell viability was determined by the MTS assay. The IC_50_ values were calculated using GraphPad Prism 6 software. **c** The kinetic effect of Zt/g4-DM1 on cell viability. H358 and H2228 cells were cultured and treated with Zt/g4-DM1 as described in **a**. Cells were collected at 24, 48, 72, and 96 h followed by the MTT assay

The effect of Zt/g4-directed DM1 delivery on cell cycle was shown in Fig. 2a. The changes in cell cycle were observed as early as 6 h after addition of Zt/g4-DM1, which features a significant reduction in G0/G1 phase, a decrease in S phase, and a dramatic increase in G2/M phase. Quantitative measurement of cell cycle changes is shown in Table 1. Clearly, Zt/g4-targeted delivery of DM1 has a profound effect on cell cycle by BC and NSCLC cells overexpressing RON.

**Table 1:** Effect of Zt/g4-Directed DM1 Delivery on Cell Cycle Changes by human breast and NSCLC Cell Lines^a^

Cancer cell lines	Changes of cell cycles after Zt/g4-DM1 treatment (%)											
	G0/G1 phase				S phase				G2/M phase			
	0 h	6 h	12 h	24 h	0 h	6 h	12 h	24 h	0 h	6 h	12 h	24 h
MCF7	50.49	51.56	47.59	42.19	23.95	23.75	25.08	26.78	25.57	24.69	27.33	31.03
DU4475	51.14	40.92	23.22	10.13	10.42	18.24	16.37	13.5	38.42	40.91	60.40	76.37
MDA-MB-231	48.38	35.40	17.56	7.23	17.52	25.21	21.90	10.05	34.24	39.50	60.63	82.62
T-47D	64.71	62.81	35.63	14.74	7.06	10.15	28.61	10.29	28.24	27.04	35.76	74.97
H1993	56.40	54.55	53.28	50.17	16.00	16.53	18.03	15.87	27.60	28.93	28.69	33.95
H2228	63.56	58.23	48.85	27.21	13.98	13.92	12.95	8.16	22.46	27.85	38.20	64.63
H358	46.60	29.22	28.13	18.69	14.63	17.81	18.23	14.65	38.78	52.97	53.65	66.67

^a^Breast and NSCLC cell lines at 1 × 106 cells per culture dish in DMEM with 10 % FBS were treated at 37 °C for 0, 6, 12 and 24 h with 5 μg/ml of Zt/g4-DM1. After treatment, cells were labeled with propidium iodide and then analyzed by an Accuri Flow Cytometer. Cell cycle changes were determined by measuring DNA contents as previously described (25)

We next studied the effect of Zt/g4-DM1 on viability of BC and NSCLC cells using the MTT assay. CmIgG-DM1 was used as the control. We first determined sensitivities of each cell line to free DM1. The calculated IC_50_ values were 98.29 ± 2.89 nM for MCF-7; 12.42 ± 2.82 nM for DU4475; 23.45 ± 3.48 nM for MDA-MB-231; 7.24 ± 1.45 nM for t-47D; 4.16 ± 0.38 nM for H1993; 7.13 ± 0.51 nM for H358; and 6.59 ± 0.44 nM for H2228, respectively. We then studied the effect of Zt/g4-DM1 on cell viability. A significant reduction in cell viability was observed in a dose-dependent manner in all tested cell lines expressing high levels of RON (Fig. 2b). The IC_50_ of Zt/g4-DM1 was 2.86 μg/ml for DU4475, 7.74 μg/ml for MDA-MB-231, 2.29 μg/ml for T- 47D, 5.13 μg/ml for H2228, and 2.44 μg/ml for H358 cells, respectively. MCF-7 cells were not sensitive to Zt/g4-DM1. Only a 12 % reduction in cell viability was observed after cells were treated with 20 μg/ml of Zt/g4-DM1. Also, H1993 cells expressing low levels of RON were only slightly sensitive to Zt/g4-DM1. A 29 % reduction in cell viability was documented after cells were treated with 15 μg/ml of Zt/g4-DM1. The kinetic effect of Zt/g4-DM1 on cell viability was studied using H2228 and H358 cells as the model (Fig. 2c). Zt/g4-DM1 caused a time-dependent reduction in cell viability in both cell lines tested. Thus, Zt/g4-DM1 is able to reduce cell viability in both dose- and time-dependent manners in cancer cells overexpressing RON.

**Fig. 3:**
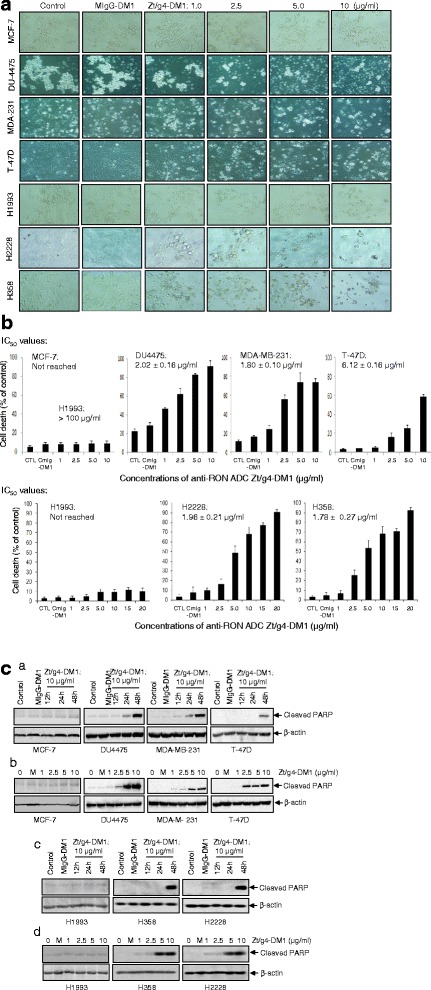
Induction of cell apoptosis by Zt/g4-DM1. **a** morphological analysis of cell death. BC and NSCLC cells were treated with different amount of Zt/g4-DM1 for 96 h. Morphological changes were observed under the Olympus BK-41 inverted microscope and photographed. Images showing cell death are presented. **b** Dose-dependent cell death. BC and NSCLC cells were cultured and treated with Zt/g4-DM1 for 96 h as described in Fig. 1. The percentages of cell death from individual cell lines were determined by the trypan blue exclusion assay. The IC_50_ values of cell death were calculated using the GraphPad Prism 6 software. **c** PARK cleavage as cellular apoptosis. BC and NSCLC cells (2 × 10^6^ cells in a 60 mm diameter dish) were treated with different amounts of Zt/g4-DM1 as described in Fig. 1. Cells were collected at different intervals. Cellular proteins (50 μg per samples) were subjected to Western blot analysis using an antibody specific to PARP fragment. Results shown here are from one of two experiments with similar results

Observation of cell morphology under a microscope indicated a massive cell death after Zt/g4-DM1 treatment in BC and NSCLC cells overexpressing RON with more than 50 % cell death after a 96 h treatment (Fig. 3a and b). The calculated IC_50_ values were 2.02 ± 0.16 μg/ml for DU4475, 1.80 ± 0.10 μg/ml for MDA-MB-231, 6.12 ± 0.16 μg/ml for T-47D, 1.78 ± 0.27 μg/ml for H358, and 1.96 ± 0.21 μg/ml for H2228 cells. Western blot analysis of the PARP activation, a cellular apoptosis indicator [38, 39] is shown in Fig. 3c. PARP fragments were evident from BC and NSCLC cells in both time and dose-dependent manners after Zt/g4-DM1 treatment. Thus, Zt/g4-DM1 not only arrests cell cycles and decreases cell viability, but also reduces viable cell numbers and induces massive apoptotic cell death.

### Correlations in vitro between the level of RON expression and the efficacy of Zt/g4-DM1 on cell viability

**Fig. 4:**
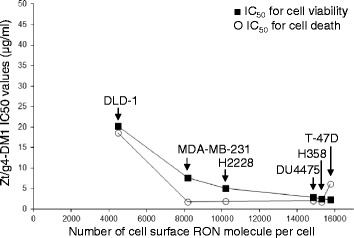
The correlation between the efficacy of Zt/g4-DM1 and the number of RON receptors expressed by cancer cells. The IC_50_ values for cell viability and cell death were calculated using the GraphPad Prism 6 software and then plotted with individual BC and NSCLC cell lines expressing different levels of RON. Colon cancer DLD-1 cells expressing low levels of RON [25] also were included for comparison. The cell surface RON numbers per cell are: DLD-1, 4480; MDA-MB-231, ~8185; H228, 10,207; DU4475, 14,841; H358, 15,286; and T-47D, 15,756 RON receptors per cell. The IC_50_ values at or less than 5 μg/ml of Zt/g4-DM1 were used as the effective dose to determine the RON receptor number required to reach the IC_95_ value. Results shown here are from one of three experiments with similar results

A comparison between the efficacy of Zt/g4-DM1 (cell viability IC_50_ value) and the number of RON receptors per cell is shown in Fig. 4. The increased efficacy of Zt/g4-DM1 as evident by decreased IC_50_ values is correlated with the number of RON molecules expressed on the cell surface. It appeared that the minimal number of cell-surface RON molecules required for Zt/g4-DM1 to achieve a 95 % reduction in cell viability (EC_95_) is around 10,000 molecules per cell. A decrease in the level of RON receptors per cell correlated proportionally with the diminished efficacy of Zt/g4-DM1. Thus, cancer cells expressing ~10,000 RON molecules on cell surface per cell appears to be required for Zt/g4-DM1 in vitro to show the maximal activity.

### Therapeutic activity of Zt/g4-directed DM1 delivery in mouse xenograft tumor model

**Fig. 5:**
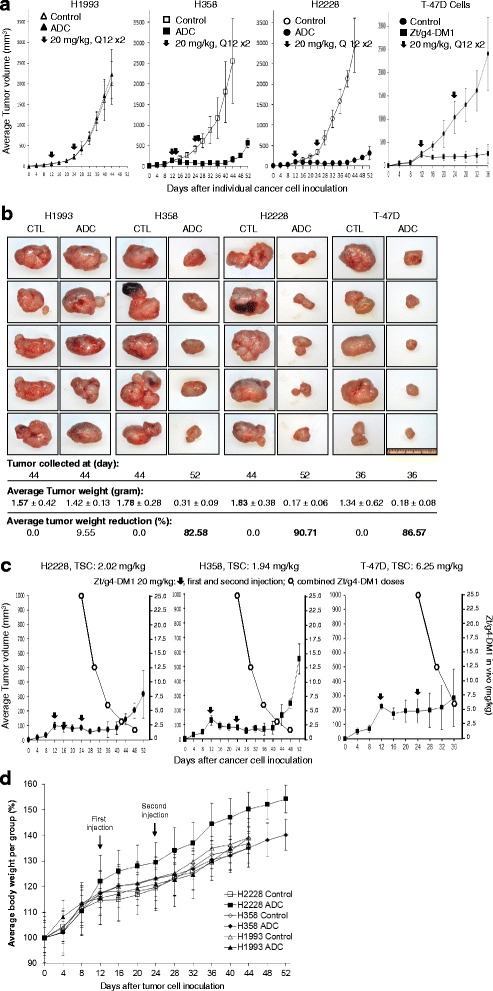
Therapeutic effect of Zt/g4-DM1 on BC and NSCLC cell-derived tumors. **a** Athymic nude mice (five mice per group) were subcutaneously inoculated with 5 × 10^6^ BC and NSCLC cells. Upon tumor volume reaching at ~100 mm^3^, Zt/g4-DM1 was injected through tail vein at 20 mg/kg in a Q12 × 2 regimen. Control mice were injected with CmIgG-DM1. Tumor growth was monitored every 4 days by measuring tumor volume. The percentages of inhibition were calculated by a formula: 1-(average tumor volume from Zt/g4-DM1 treated group)/(average tumor volume from control mice) × 100 %. **b** Individual tumors from the control and experimental groups were collected and weighed at day 36, 44, or 52 dependent on individual tumor growth curves. The percentages of average tumor reduction were calculated by a formula: 1- (average tumor weight from Zt/g4-DM1 treated group)/(average tumor weight from control mice) × 100 %. **c** The TSCs of Zt/g4-DM1 in vivo. The growth curve of individual xenograft tumors from BC or NSCLC was analyzed with dynamics of Zt/g4-DM1 at 20 mg/kg in the Q12 × 2 regimen. Zt/g4-DM1 in vivo has a terminal half-life (t½) of 6.01 days [25], which was plotted to the tumor regrowth curve. TSCs for H2228, H358, and T-47D cell xenograft models were determined as the minimal dose of Zt/g4-DM1 required to balance the tumor growth and inhibition. **d** The effect of Zt/g4-DM1 on mouse body weight. Control and tumor-bearing mice after Zt/g4-DM1 treatment as described in A were monitored every 4 days for changes in their body weights. The percentages of changes in mouse body weight were calculated as previously described [25]

The goals of the in vivo study were to validate the efficacy of Zt/g4-DM1 and to determine the tumoristatic concentration (TSC). The TSC is a dose that reaches a balance in vivo between the tumor growth and inhibition. To this end, mouse xenograft tumor models of H1993, H288, H358, and T-47D cells were established. Xenograft models for MDA-MB-231 and DU4475 cells were unable to be established. Zt/g4-DM1 at 20 mg/kg with a Q12 × 2 schedule was used for in vivo studies, which was designed according to our previous studies and the terminal half-life (t½: 6.01 days) of Zt/g4-DM1 (25). As shown in Fig. 5a, xenograft tumors initiated by H1993 cells expressing only 2152 RON receptors per cell were insensitive to Zt/g4-DM1. In contrast, Zt/g4- DM1 was highly effective in delaying tumor growth caused by H2228, H358 and T-47D cell lines. The effect of Zt/g4-DM1 in vivo was long lasting. At day 40, the tumor growth inhibition was 96.12 % for H2228 and 95.66 % for H358 cell xenograft tumors. For T-47D cell-initiated tumors, Zt/g4-DM1 showed a tumor growth inhibition at 86.4 % at day 36.

A comparison of tumor weight between the control and Zt-g4-DM1 treated group at the end of experiments was shown in Fig. 5b. In Zt/g4-DM1-treated H1993 xenograft tumors, we did not observe a statistical difference in tumor weight between the control and experimental group, confirming that Zt/g4-DM1 has no effect on tumors expressing low levels of RON. In contrast, Zt/g4-DM1 was effective in inhibition of tumors expressing high levels of RON. Although tumors from Zt/g4-DM1-treated H2228 and H358 cell groups were collected 8 days later (day 44 for control mice versus day 52 for experimental animal), a significant reduction in average tumor weight was still observed for H358 (82.58 %) and H2228 (90.71 %) cell-derived xenograft tumors. Similarly, significant reduction in average tumor weight (86.57 %) also was documented in T-47D cell derived xenograft tumors. Thus, Zt/g4-DM1 at 20 mg/kg in the Q12 × 2 regimen is effective in inhibition of xenograft tumor growth initiated by H2228, H358 and T- 47D cells.

We calculated the TSCs based on the terminal half-life of Zt/g4-DM1 in relationship with the tumor regrowth curve (Fig. 5c). Zt/g4-DM1 in vivo has a terminal half-life (t½) of 6.01 days [25]. The regrowth was found at day 44 for both H2228 and H358, and day 36 for T-47D- xenografts. The calculated TSCs were 2.02 mg/kg for H2228, 1.94 mg/kg for H358, and 6.25 mg/kg for T-47D cell-derived xenograft tumors, respectively. These results indicate that in vivo Zt/g4-DM1 at ~2 mg/kg is required to reach a tumoristatic status for H2228 and H358 cell-derived xenograft tumors. However, a relatively high TSC at ~6.25 mg/kg is necessary to balance the T-47D cell-derived tumor growth and inhibition.

To exclude any possibilities that reduction of tumor is related to general toxicity of Zt/g4- DM1 in vivo, we monitored body weight of individual tumor-bearing mice before and after treatment (Fig. 5d). All mice behaved normally and their body weight progressively increased during the entire observation period. Injection of cancer cells did not alter mouse growth curve. Significantly, Zt/g4-DM1 treatment did not halt mouse growth with increased body weight. The average body weight of experimental groups was comparable to that of control mice with no statistical differences. Thus, results in Fig. 5d demonstrate that tumor reduction is not related to the general toxicity of Zt/g4-DM1 on mouse growth.

### Therapeutic effect of Zt/g4-DM1 in combination with chemotherapeutics

**Fig. 6:**
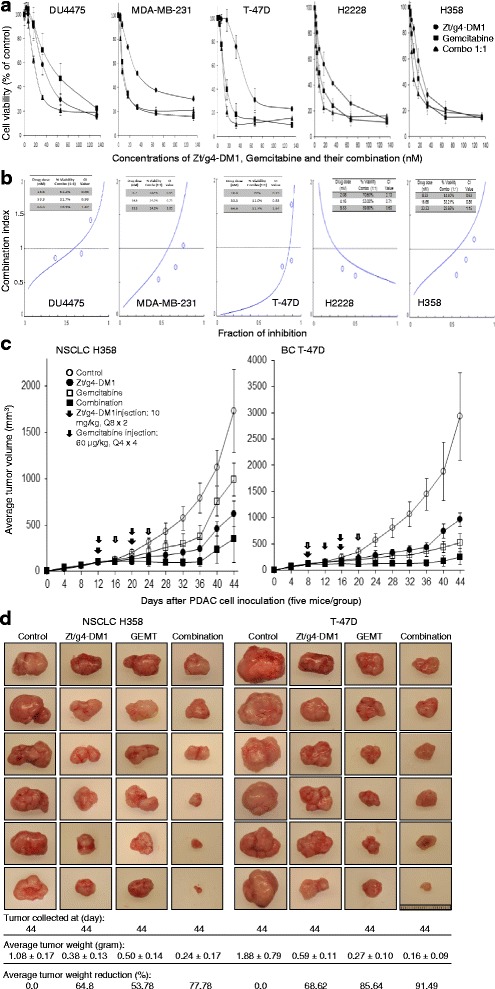
Synergism between Zt/g4-DM1 and chemotherapeutics. **a** the synergistic effect of Zt/g4-DM1 and gemcitabine in vitro. BC and NSCLC cell lines (8000 cell per well in a 96-well plate in triplicate) were cultured in DMEM with 10 % FBS and treated for 96 h with different amounts of gemcitabine, Zt/g4-DM1, or their combinations at 1:1 molar ratio. Cell viability was measured using the MTT assay. **b** Analysis of the synergism by Chou-Talalay plot. Percentages of cell viability from individual samples as described in A were calculated, converted, and then used for the fraction of inhibition-combination index (CI) plot as previously described [43]. Data shown here are from one of three experiments with similar results. **c** Effect of Zt/g4-DM1 in combination with gemcitabine in vivo. Athymic nude mice (five mice per group) were subcutaneously inoculated with 5 × 10^6^ H358 or T-47D cells to allow tumor growth to reach an average volume at ~100 mm^3^. Zt/g4-DM1 at 10 mg/kg in a Q8 × 3 regimen was injected through tail vein. Mice injected with CmIgG-DM1 were used as the control. Gemcitabine was injected into the intraperitoneal cavity at 60 mg/kg in a Q4 × 4 schedule. Both drugs were used for the combination group according to their own dose and schedule. Tumor volume was measured every 4 days. The percentages of tumor growth inhibition were calculated from the average tumor volume as described in Fig. 5a. **d** Tumor inhibition by measuring tumor weight. Individual tumors from different groups were collected and weighed to obtain the average tumor weight (gram). The percentages of reduction in average tumor weight were calculated as described in Fig. 5b

We first studied the activities of Zt/g4-DM1 in combination with chemotherapeutics. BC and NSCLC cell lines were treated for 96 h in vitro with different amounts of gemcitabine, Zt/g4-DM1 or their combinations at 1 to 1 molar ratio. Results in Fig. 6a show a progressive reduction of cell viability in both single and combination-treated groups in all five cancer cell lines tested. Analysis of data by plotting the fraction of inhibition against the combination index (Fig. 6b) confirmed a synergism between Zt/g4-DM1 and gemcitabine at the defined concentrations in five cancer cell lines tested. An interesting finding was in H2228 cells, in which a synergistic activity was only observed when the low concentration of two drugs were used (Fig. 6b).

We then performed the combination therapy in mouse xenograft tumors, in which Zt/g4- DM1 at 10 mg/kg in the Q8 × 2 regimen was combined with gemcitabine at 60 mg/kg in the Q4 × 4 schedule (Fig. 6c). Gemcitabine alone partialy inhibited H358 and T-47D xenograft tumors in a time-dependent manner with an average reduction in tumor volume at 51.3 and 73.8 % at day 36, respectively. Similarly, Zt/g4-DM1 alone also partially delayed the H358 and T-47D xenograft tumor growth at day 36 with an average 68.0 and 71.1 % reduction in tumor volume, respectively. The combination therapy resulted in a significant inhibition of H358 and T-47D xenograft tumors. An average growth inhibition in tumor volume with statistical difference (*p*: <0.05) was 88.43 % for H358 and 90.62 % for T-47D. Measurement of tumor weight at the end of the study further confirmed an increase in inhibition of tumor growth. For H358 xenograft tumors, the average tumor weight from the combined treatment groups (0.24 g ± 0.17) was significantly less than that from the Zt/g4-DM1-treated group (0.38 g ± 0.13, *p* <0.05) and from the gemcitabine-treated group (0.50 g ± 0.14, *p* <0.05) (Fig. 6d). Similar results also were observed in T-47D xenograft tumors, in which the average tumor weight from the combination group (0.16 g ± 0.09) was much less than Zt-g4-DM1 (0.59 g ±0.11) or gemcitabine (0.27 g ± 0.10) treated groups (*p* <0.05). Thus, Zt/g4-DM1 in combination with gemcitabine results in the increase in inhibition of T-47D and H358 xenograft tumors.

## Discussion

The study presented here shows the effectiveness of Zt/g4 in RON-targeted DM1 delivery for treatment of BC and NSCLC. Both in vitro and in vivo studies demonstrated the capability of Zt/g4 in induction of RON internalization by cancer cells. Also, Zt/g4-directed delivery of DM1 is sufficient to cause cell cycle arrest in G2/M phase, to inhibit cell proliferation and to cause massive cell death. These functional analyses help to establish a correlation between the IC_50_ values for cell viability and the levels of RON expression by cancer cells. Studies from mouse xenograft tumor models confirmed that Zt/g4 is suitable for DM1 delivery in vivo and Zt/g4-DM1 at 20 mg/kg in the Q12 × 2 regimen inhibits tumor growth with a long-lasting effect. The combinational therapy further demonstrated that Zt/g4-DM1 together with chemoagents results further in delaying BC and NSCLC xenograft tumor growth. Thus, Zt/g4-DM1 is effective in the model of anti-RON ADC for treatment of BC and NSCLC overexpressing the RON receptor tyrosine kinase.

Selection of Zt/g4 for DM1 conjugation and delivery is based on its unique feature in induction of RON internalization [25, 34]. We noticed that the kinetics of Zt/g4—induced RON internalization between BC and NSCLC cell lines are quite different. The IE_50_ values for BC cell lines expressing the high levels of RON (more than 8000 RON molecules per cell) is in the range of 10 ~ 16 h. In contrast, the IE_50_ values for NSCLC H2228 and H358 cell lines expressing similar levels of RON were at the 7 to 10 h ranges. The mechanism(s) underlying such differences currently is unknown. Regardless of the difference, Zt/g4-induced RON internalization is highly effective with more than 85 % of cell surface RON internalized within 36 h. In the case of H358 cells expressing ~15,000 RON molecules per cell, it translates into 12,750 RON receptors that are internalized within 36 h. This is equivalent to 51,000 DM1 molecules delivered into a single cell, which is sufficient to cause cell cycle arrest. In cell lines such as H1993 expressing the low level of RON, Zt/g4 was able to induce RON internalization with IE_50_ similar to those of other cancer cell lines (Fig. 1b). However, the amount of internalized payload is significantly less by calculation due to the low levels of the RON expression. Considering the fact that normal epithelial cells express low levels of RON, we reason that the low density of RON in normal epithelial cells is not enough to bring sufficient amount of DM1 to cause significant biological activity. Results in Fig. 2 appear to support this notion. Thus, Zt/g4-induced RON internalization facilitates intracellular delivery of DM1 in cancer cells expressing high levels of RON.

The action of DM1 delivered by Zt/g4 was displayed in both BC and NSCLC cells. First, we showed by flow cytometric analysis that the Zt/g4-DM1 internalization causes cell cycle arrest in G2/M phase, which is a feature of DM1 that impairs microtubule dynamics [40]. This effect was observed as early as 6 h after the addition of Zt/g4-DM1 and characterized by progressive reduction of the G1 phase and the accumulation of cells at the G2/M phase. Second, we found that Zt/g4-directed DM1 delivery progressively decreases cell viability. More than 80 % reduction in cell viability 96 h after treatment was achieved among three BC and two NSCLC cell lines tested. Third, we documented a massive cell death in Zt/g4-DM1-treated BC and NSCLC cells in a dose-dependent manner with Zt/g4-DM1 IC_50_ values ranging from 2 to 6 μg/ml. Analysis of cellular markers for apoptotic death further confirmed that Zt/g4-DM1 treatment activates PARP as evident by the presence of the PARP fragment. These findings indicate that DM1-mediated cell cycle arrest leads to initiation of an intracellular apoptotic pathway. Finally, we discovered that the efficacy of Zt/g4-DM1 in vitro directly correlates with the levels of the RON expression by cancer cells. For Zt/g4-DM1 to achieve a significant reduction in cell viability, the minimal RON molecule expressed by cancer cells should be at ~8000 receptors per cells. We reason that such a correlational relationship is useful for predicting the in vitro effectiveness of Zt/g4-DM1 against cancer cells, which helps to select potential target cancer cells for evaluation in vivo.

Results from mouse xenograft tumor models demonstrate the efficacy of Zt/g4-DM1 in inhibition of tumor growth. It is possible that Zt/g4-mediated immunological activities may also contribute the observed therapeutic efficacy. As described in other ADCs, antibody-dependent cell-mediated cytotoxicity, antibody dependent cellular phagocytosis, and complement- dependent cytotoxicity play a role in vivo in the ADC anticancer activity [13]. We used Zt/g4- DM1 at 20 mg/kg in the Q12 × 2 regimen for the animal study. This dosing regimen was designed to determine whether Zt/g4-DM1 at a total of 40 mg/kg in a particular period is sufficient to inhibit tumor growth. We showed that the action of Zt/g4-DM1 is dependent on levels of the RON expression. Zt/g4-DM1 effectively inhibited xenograft tumor growth mediated by H228, H358, and T-47D cells but has no effect on H1993 cell-mediated tumors. By applying pharmacokinetic data of Zt/g4-DM1, we monitored Zt/g4-DM1 efficacy for several half-life cycles, which confirms that Zt/g4-DM1 has the long-lasting effect of up to 2 weeks without signs of tumor regrowth (from day 24 to day 40 in the NSCLC model and from day 24 to day 36 in the BC model) (Fig. 5a). Moreover, we established the TSC as the indicator of Zt/g4-DM1 for monitoring xenograft tumor growth, which should help us to predict the dose-response curve and to design the treatment regimen for further study [41, 42]. Finally, we observed that the Zt/g4-DM1 regimen used in vivo did not exert significant toxicity towards animals. An increase in the average body weight was observed in Zt/g4-DM1 treated animals. Considering these facts, we conclude that Zt/g4-DM1 is effective and safe in the targeted treatment of BC and NSCLC xenograft tumors.

Studies using H358 and T-47D xenograft models demonstrated the increase in therapeutic activity between Zt/g4-DM1 and gemcitabine in inhibition of tumor growth. We showed from in vitro studies that the combination of Zt/g4-DM1 with chemotherapeutics at the same molar ratio further reduce viability of BC and NSCLC cells. Analysis of the average tumor weight at the end of the study also confirmed the increase in therapeutic activity between Zt/g4-DM1 and chemotherapeutics. This indicates that the inhibition can be synergized through different mechanisms of action. It is worth to note that the synergism of Zt/g4-DM1 with gemcitabine is dependent on individual cancer cell lines in the particular dose range. We reasoned from the combination therapy that since BC and NSCLC tumors are highly malignant at the later stage, Zt/g4-DM1 in combination with chemotherapeutics could be a strategy for targeted cancer therapy.

## Conclusions

We present evidence in this study that anti-RON antibody directed delivery of the highly potent drug DM1 in the form of ADC is a therapeutic strategy for targeted treatment of breast and NSCLC cancer in animal models. Results from various in vitro and in vivo experiments showed that Zt/g4-DM1 not only significantly decreases viability of breast cancer and NSCLC cells but also dramatically delays the tumor growth in mouse xenograft tumor models. Moreover, we demonstrated that Zt/g4-DM1 in combination gemcitabine significantly increases the inhibitory effect on xenograft tumor growth. These findings lay the foundation for clinical development of anti-RON ADC Zt/g4-DM1 for potential cancer therapy.

## Abbreviations

ADC: antibody-drug conjugate
ADCC: antibody-dependent cell- mediated cytotoxicity
ADCP: antibody-dependent cellular phagocytosis
ATCC: American type cell culture
BC: breast cancer
CDC: complement-dependent cytotoxicity
DAPI: 4′,6-diamidino-2- phenylindole
DM1: N2′-deacetyl-N2′-(3-mercapto-1-oxopropyl) maytansine
FITC: fluorescein isothiocyanate
HIC: hydrophobic interaction chromatography
LAMP-1: lysosomal-associated membrane protein-1
LY6E: lymphocyte antigen 6 complex, locus E
mAb: monoclonal antibody
NSCLC: non-small cell lung cancer
RON: Recepteur d’Origine Nantais
PARP: poly ADP ribose polymerase
SMCC: N-succinimidyl-4-[maleimidomethyl]-cyclohexane carboxylate
TKI: tyrosine kinase inhibitor
TMA: therapeutic monoclonal antibody
TSC: tumoristatic concentration
