# Erratum to: Biological evaluation of antibody-maytansinoid conjugates as a strategy of RON targeted drug delivery for treatment of non-small cell lung cancer

**DOI:** 10.1186/s13046-016-0364-5

**Published:** 2016-06-17

**Authors:** Liang Feng, Hang-Ping Yao, Sharad Sharma, Yong-Qing Zhou, Jianwei Zhou, Ruiwen Zhang, Ming-Hai Wang

**Affiliations:** State Key Laboratory for Diagnosis & Treatment of Infectious Diseases and Collaborative Innovation Center for Diagnosis & Treatment of Infectious Diseases, First Hospital of Zhejiang University School of Medicine, Zhejiang, China; Department of Biomedical Sciences, Texas Tech University Health Sciences Center School of Pharmacy, 1406 Coulter Street, Suite 1117, Amarillo, TX 79106 USA; Department of Neurosurgery, First Hospital of Zhejiang University School of Medicine, Zhejiang, China; Department of Molecular Cell Biology & Toxicology, Nanjing Medical University School of Public Health, Jiangsu, China; Department of Pharmaceutical Sciences, Texas Tech University Health Sciences Center School of Pharmacy, Amarillo, TX USA

## Erratum

Unfortunately, the original version of this article [1] contained an error. The name of one of the authors, Sharad Sharma, was not included in the author list.

The correct author list and the new “Authors’ contributions” section have been correctly included in full in this erratum and updated in the original article.
